# Exploring Professional Experiences in Caring for Vulnerable Migrants in an Italian Rural Reception Centre: A Qualitative Study Using Multidimensional Textual Analysis—Professional Experiences in Rural Migrant Care

**DOI:** 10.1002/nop2.70757

**Published:** 2026-08-30

**Authors:** Mariachiara Figura, Gianluca Pucciarelli, Silvio Simeone, Paola Arcadi, Izabella Uchmanowicz, Ercole Vellone, Rosaria Alvaro

**Affiliations:** ^1^ Department of Maternal and Child Health Promotion, Internal Medicine & Specialties of Excellence “G. D’Alessandro” (PROMISE) University of Palermo Palermo Italy; ^2^ Department of Biomedicine and Prevention University of Rome Tor Vergata Rome Italy; ^3^ Department of Experimental and Clinic Medicine University of Catanzaro Magna Grecia Catanzaro Italy; ^4^ ASST Melegnano e della Martesana Cernusco Sul Naviglio Italy; ^5^ Division of Research Methodology, Department of Nursing, Faculty of Nursing and Midwifery Wroclaw Medical University Wroclaw Poland

**Keywords:** intercultural competencies, multidisciplinary teams, professional well‐being, vulnerable migrants

## Abstract

**Aim:**

This study aims to explore the experiences, strengths, challenges, and potential improvements for professionals in managing the complex needs of vulnerable migrants (VM) in an Italian rural reception centre.

**Methods:**

A qualitative study using semi‐structured interviews was conducted in April 2024. Data were analysed using the Automatic Analysis of Textual Data, based on Fraire's seven‐step model for Exploratory Multidimensional Data Analysis.

**Data Sources:**

Data were collected from 16 professionals working in a rural reception centre in southern Italy. Interviews were conducted and analysed using AATD in April 2024.

**Findings:**

The analysis identified two main dimensions of professionals' roles: balancing systemic responsibilities with personal engagement and managing immediate needs versus long‐term integration goals. Professionals face significant challenges, such as resource scarcity, bureaucratic inefficiencies, and emotional fatigue, which impact their well‐being and the quality of care provided to migrants. Resilience, adaptability, and multidisciplinary collaboration were identified as key strengths.

**Conclusion:**

The study highlights the dual nature of professionals' work in reception centres, requiring them to balance operational tasks with emotional involvement in migrant care. Targeted interventions and systemic reforms are necessary to support professionals and enhance the quality of care for vulnerable migrants, particularly in resource‐constrained rural settings.

**Implications for Practice and/or Patient Care:**

This study underscores the importance of providing targeted support to professionals working in reception centres, including training in intercultural competence, stress management, and coping strategies. Policies should address systemic challenges and provide resources to enhance healthcare delivery and social integration programs.

**Reporting Method:**

This study adhered to the EQUATOR guidelines for reporting qualitative research (COREQ). The findings were reported in compliance with these guidelines, ensuring methodological rigour and transparency.

**Patient or Public Contribution:**

No patient or public contribution.

**Implications for the Profession and/or Patient Care:**

This study highlights the critical need for targeted support and training for professionals working in reception centres, particularly in rural settings. To improve care for vulnerable migrants, professionals should receive training in intercultural competence, stress management, and coping strategies to better navigate the complex challenges they face. Furthermore, systemic changes are necessary to alleviate the pressures on reception centres, such as streamlining bureaucratic processes and enhancing healthcare infrastructure, particularly in rural areas where resources are limited. By addressing these needs, we can improve the well‐being of both the professionals and the migrants they serve, fostering more effective support systems and better care outcomes. Additionally, fostering multidisciplinary collaboration and community engagement can contribute to more comprehensive and sustainable care models.

**Protocol Registration:**

The Ethics Committee of the University of Rome Tor Vergata approved this study on 07/07/2021 (protocol registration number 160.21).

## 1 Introduction

In recent decades, international migration to Europe has increased substantially, driven by armed conflicts, political instability, economic inequalities, and environmental crises. These dynamics have exposed migrants to multiple forms of vulnerability along migratory routes and after arrival in host countries, making migration one of the most complex public health and social challenges of contemporary societies (WHO 2024). Southern European countries, including Italy, have become key points of arrival and reception, necessitating the organization of structured reception systems that can respond to migrants' health, social, and legal needs (Department of Civil Liberties and Immigration 2024; Frontex 2024).

Migrants, hereafter referred to as Vulnerable Migrants (VM), often arrive with a complex combination of physical, psychological, and social vulnerabilities shaped by pre‐migration trauma, perilous journeys, and post‐migration stressors (World Health Organization 2023). Although the so‐called ‘healthy migrant effect’ has been described in the early phases of migration (Fennelly 2007; Yu and Teschke 2018), this advantage frequently diminishes over time due to chronic stress, social exclusion, language barriers, and limited access to healthcare. These conditions increase the risk of mental health problems, chronic diseases, and social marginalization, particularly among migrants hosted in reception centres (Mancini et al. 2019; SPRAR 2016). As a result, reception systems are required to provide not only basic assistance but also integrated and culturally responsive care pathways.

Within this context, healthcare and social professionals play a pivotal role in ensuring continuity of care and supporting migrants' integration processes. Multidisciplinary teams, composed of nurses, physicians, psychologists, social workers, educators, and administrative staff, are required to address highly heterogeneous needs, often under conditions of limited resources and organizational constraints (Debesay et al. 2022; Mancini et al. 2019; Pettersen and Debesay 2023). Among these professionals, nurses frequently represent the first point of contact with health services and act as key mediators between migrants, healthcare systems, and local communities. Their role extends beyond clinical care to include health education, coordination of services, emotional support, and cultural mediation (Badanapurkar et al. 2025; Harris et al. 2024).

However, the professional–migrant caring relationship is characterized by significant transcultural challenges. Differences in language, health beliefs, expressions of suffering, and expectations of care can complicate communication and mutual understanding, increasing the risk of misunderstandings and emotional strain (Gaya‐Sancho et al. 2021; Rissel et al. 2023). Nurses and other professionals are therefore required to navigate complex ethical dilemmas, balance institutional rules with individualized care, and manage the emotional impact of working with people who have experienced trauma, loss, and displacement (Debesay et al. 2022; Gemignani and Giliberto 2021). These demands call for high levels of cultural competence, reflexivity, and emotional resilience, particularly in contexts where professional support structures are limited (Wilson et al. 2022).

International literature has consistently shown that working with refugees and forcibly displaced populations exposes healthcare professionals and volunteers to substantial emotional and organizational demands. A recent systematic review by Roberts et al. (2021) reported pooled prevalence rates of 29.7% for high‐level burnout and 45.7% for moderate‐to‐severe secondary traumatic stress among professionals and volunteers working with forcibly displaced people. These findings are further supported by Mavratza et al. (2021), who identified a high risk of compassion fatigue in 25.6% of participants involved in refugee support. In addition to emotional burden, previous studies have highlighted structural and relational challenges that complicate care delivery. Kavukcu and Altıntaş (2019) identified linguistic and cultural barriers, limited professional knowledge, and inefficiencies in healthcare systems as key obstacles in caring for migrant populations. More recently, Ghafoori et al. (2024) emphasized the pervasive impact of vicarious traumatization and difficulties in coping among professionals repeatedly exposed to refugees' traumatic experiences. These findings support the need for targeted organizational strategies, professional training, and psychological support to promote workforce sustainability and quality of care in migrant reception settings.

Such challenges are often amplified in rural reception centres, where geographic isolation, limited healthcare infrastructure, and reduced access to specialized services place additional pressure on professionals (Arcadi et al. 2024; Dong et al. 2023; Serre‐Delcor et al. 2021). In these settings, staff are frequently required to compensate for structural deficiencies through personal involvement, creativity, and informal networks, further blurring the boundaries between professional roles and relational engagement. Despite the growing recognition of these dynamics, existing research has predominantly focused on migrants' perspectives, while the lived experiences of professionals working in reception centres, particularly in rural contexts, remain underexplored.

Moreover, few studies have adopted analytical approaches capable of capturing the multidimensional nature of professionals' experiences, which encompass organizational demands, relational dynamics, emotional labor, and long‐term integration goals. Understanding how professionals construct meaning around these tensions in their everyday practice is essential to inform supportive interventions, improve workforce sustainability, and enhance the quality of care provided to vulnerable migrant populations (Figura et al. 2024, 2025).

Against this backdrop, the present study aims to explore the experiences, strengths, challenges, and perceived areas for improvement among professionals working in an Italian rural reception centre for vulnerable migrants. By applying a multidimensional textual analysis based on a qualitative approach, this study seeks to uncover latent dimensions within professionals' narratives, offering a comprehensive understanding of how systemic responsibilities and personal engagement intersect in everyday care practices.

### 1.1 Aim

This study aimed to explore the lived experiences of professionals working in an Italian rural reception centre in caring for VM.

## 2 Methods

### 2.1 Design

A qualitative study with semi‐structured face‐to‐face interviews was conducted. Data analysis was performed by using the AATD following the methodological framework developed by Fraire (2009). The Consolidated Criteria for Reporting Qualitative Research (COREQ) were followed in writing the research report (Tong et al. 2007). The completed COREQ checklist is provided as Data S1.

### 2.2 Sampling and Data Collection

The enrolment of participants involved in the welcoming project was achieved using a purposive sampling approach. Data were collected at a reception centre located in southern Italy in April 2024. Individual semi‐structured interviews were conducted, recorded digitally, and stored. Questions aimed to explore participants' experiences, strengths, challenges, and potential improvements in their work. It also delves into their personal reflections on the meaning of *being a migrant* within this context (Table 1).

**TABLE 1:** Interview guide.

Interview guide
Can you share your experience as a professional in this welcoming centre?
What are the strengths and challenges of the work you do in Camini?
If you could change something, what would it be?
Considering your work in Camini, what does ‘being a migrant’ mean to you?

An empathetic approach was maintained throughout the interviews to facilitate the stories of the participants. The interviews continued until data saturation, as defined by Corbin and Strauss (1998), was reached. The average duration of the interview was 40 min. In addition, a short questionnaire was administered to collect sociodemographic data from the participants. These data contributed to the multidimensional analysis and provided an overview of the characteristics of the sample.

### 2.3 Data Analysis

Data analysis was conducted by a researcher specialized in AATD (MF). It is a data analysis technique designed to identify hidden patterns, themes, and relationships within qualitative data. AATD allows for the identification of similarities and differences in language use across interviews, providing insights into the deeper structure of the discourse. The process followed Fraire's seven‐step model for Exploratory Multidimensional Data Analysis (EMDA) (Figure S1).

The interviews were conducted in Italian and then translated into English to facilitate international dissemination and publication. The translation process adhered to the cross‐cultural adaptation principles (Chen and Boore 2010). The translated interviews were then compiled into a single file, referred to as the ‘corpus’, and the ‘*a* priori *coding*’ process, which prepares the data before submission to the software, was initiated.

In the a priori coding, each interview was divided into text segments, with each segment corresponding to a specific interview question. Metadata lines, containing information about the speaker and the question domain, were placed above each segment (Table S1). Metadata were essential during statistical analysis, because allowed to choose the variable guiding the analysis. The question domain was identified through a coding process, as shown in Table 2, resulting in four domains: (a) ‘*experience*’; (b) *‘strengths and barriers*’; (c) ‘change’; and (d) ‘being a migrant’. Then, the document was submitted to the software, and the a posteriori *coding* was performed.

**TABLE 2:** Question domains according to the interview guide.

Interview guide	Question domain
What inspired you to start this journey, and can you share a significant experience from your time in Camini that shaped your perspective or approach?	Experience
What are the strengths and challenges of the work you do in Camini?	Strength and barriers
If you could change something, what would it be?	Change
Considering your work in Camini, what does ‘being a migrant’ mean to you?	Being a migrant

Then, the a posteriori *coding phase* aimed to examine the data more deeply, before statistical analysis. By creating data matrices, some indices to verify text reliability and consistency were calculated (*lexical balance*). According Bolasco (2021), the analysis required a minimum of 25,000 occurrences, meaning that the sum of all word instances, including repetitions, in the corpus must exceed 25,000 to ensure the reliability of the analysis. Additionally, the ratio of unique words to total words should be under 20% (*Type‐token ratio—*TTR), and no more than 50% of the words should appear only once in the corpus (referred to as ‘*hapax*’). These criteria were essential to ensure the reliability of the analysis. Once the criteria were verified, statistical analyses, like Multiple Correspondence Analysis (MCA), Hierarchical Descendant Classification (HDC), and Principal Component Analysis (PCA) were conducted.

MCA examined relationships among categorical variables and text segments. In our study, the categorical variable was the question domain. HDC, an unsupervised clustering method, grouped text segments with similar linguistic features, facilitating structured classification. PCA reduced the dimensionality of numerical data, enabling the identification of latent themes by extracting key components, thus synthesizing large textual datasets. The final step included generating tables and graphical outputs for visualization (Figura et al. 2023). IRaMuTeQ 0.7 alpha 2 open source was used as software for data analysis.

Sociodemographic data were analysed with descriptive statistics (mean, standard deviation).

### 2.4 Rigour and Reflexivity

According to Lincoln and Guba's criteria, methodological rigour and reliability were ensured (Lincoln 1995). *Bracketing* was performed by documenting researchers' ideas and preconceptions about the phenomenon before data collection, preventing biases influencing the analysis (Tufford 2012). Credibility was further established through researcher triangulation, where two independent researchers analysed the interviews and were improved by group discussions to reach a consensus on interpretations. Member checking was performed to seek participant confirmation of results, promote critical reflection, and minimize bias. The validity of the computer‐statistical analysis was ensured through preliminary descriptive analysis, demonstrating consistency in the text for statistical reliability. Furthermore, the use of software to analyse qualitative data improved accuracy, increased rigour, and controlled data manipulation, addressing historical shortcomings in qualitative research (Figura et al. 2023). According to Fraire (2009), the rigour of the chosen statistical analysis technique and the use of software limited the manipulation of variables to their choice.

### 2.5 Ethical Considerations

The Ethics Committee of the University of Rome Tor Vergata approved this study on 07/07/2021 (protocol registration number 160.21). To protect privacy, all respondents signed an informed consent form according to the responsibilities provided by the rules of good clinical practice and in full compliance with current legislation on personal data protection (World Medical Association 2013). The ethical standards and guidelines for the research project were respected and confidentiality was guaranteed. All participants gave their consent to the processing of data for research purposes.

## 3 Findings

### 3.1 Sample Characteristics

The sample consisted of sixteen stakeholders involved in a reception project. Among them, six were healthcare professionals (37.5%), six worked in the socioeducational field (37.5%), and four were in the organizational field (25%). The sample was predominantly male (68%) with an average age of 41 years (SD ±11.5). Most stakeholders had education beyond high school, and 75% had completed specialized training in a multicultural field. The participants had worked in Camini for an average of 6 years (SD ±5) (Table 3).

**TABLE 3:** Sample characteristics.

I.D	Gender	Age	Ed. level	Marital status	Job occupation	Year of work exp	Year of work with migrants	Multicult training
Yb15	F	30	High School	Single	Teacher	6	6	No
Az01	M	44	High School	Married	Project Director	20	10	Yes
Bv02	F	43	High School	Married	Project Coordinator	8	8	Yes
Xc16	F	42	Master's Degree	Married	Cultural Mediator	10	10	Yes
Hp08	F	38	Master's Degree	Married	Ethnopsycologist	10	2	Yes
Za14	F	34	Bachelor's Degree	Married	Nurse	7	1	No
Xy13	F	66	Bachelor's Degree	Single	General Doctor	38	13	No
Lw12	M	33	Master's Degree	Married	Psychologist	9	4	Yes
Cu03	F	33	Bachelor's Degree	Married	Psychologist	8	4	Yes
Jn10	M	68	Master's Degree	Married	Specialist Doctor	41	6	No
Io09	F	32	Bachelor's Degree	Married	Legal Operator	2	3	Yes
Km11	F	34	Middle School	Married	Home Care Worker	7	7	No
Gq07	F	34	Master's Degree	Single	Educator	7	7	Yes
Fr06	F	32	Bachelor's Degree	Married	Social Operator	7	7	Yes
Es05	M	45	High School	Married	Teacher	20	5	Yes
Dt04	M	48	Bachelor's Degree	Single	Social Operator	15	3	Yes

### 3.2 Corpus Description

The corpus analysed consisted of sixteen interviews and 64 text segments. The lexical balance showed a corpus with a rich vocabulary (Type/Token ratio: 9.95%; Hapax: < 50%) and suitable for multidimensional statistical analysis (29.025 occurrences) (Table 4).

**TABLE 4:** Lexical balance.

Number of texts	62
Number of occurrences	29,025
Number of forms	2888
Number of hapaxes	1220 (4.20% delle occorrenze − 42.24% delle forme)
Average occurrences per text	468.15

### 3.3 Multidimensional Analysis

#### 3.3.1 Correspondence Analysis

**FIGURE 1:**
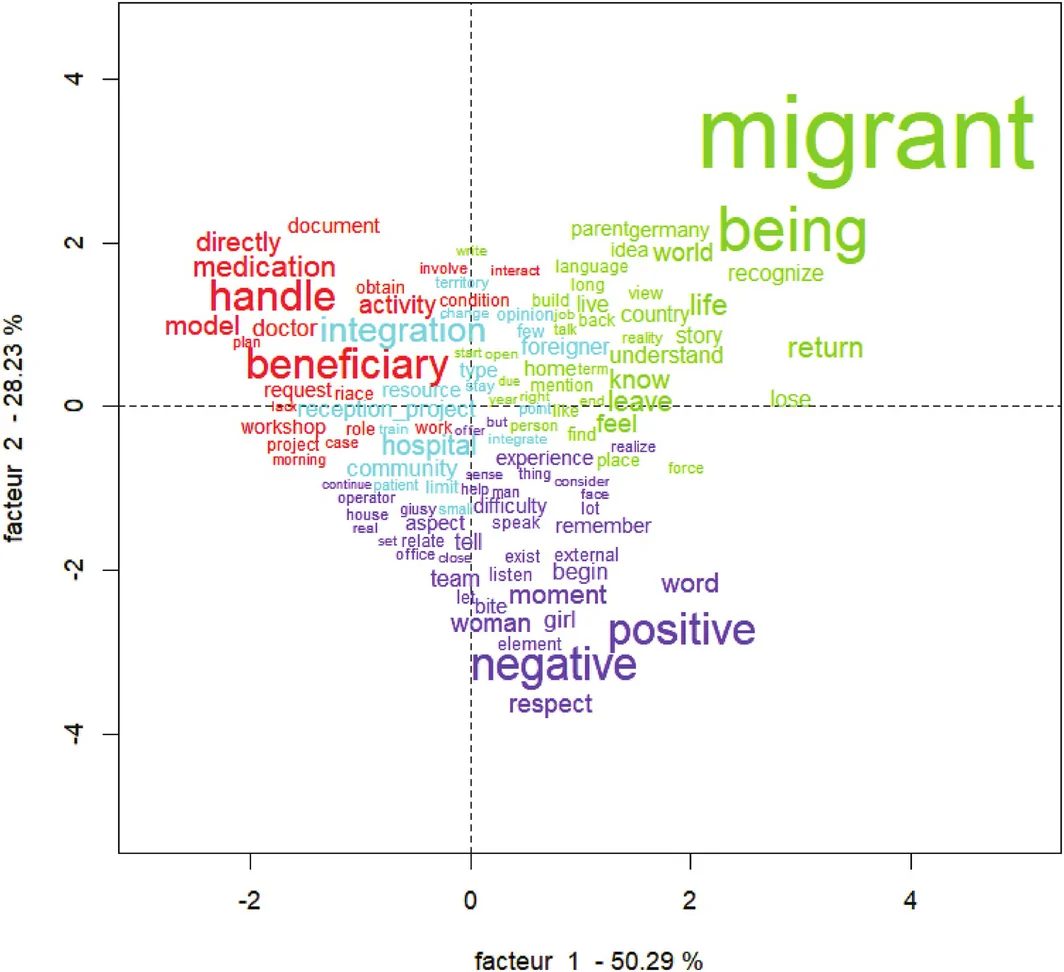
Correspondence analysis according to the question domain.

Correspondence analysis revealed a four‐quadrant graph representing the distribution of keywords based on the themes of the questions asked during the interviews, resulting in four clusters of words (Figure 1). The clusters' positioning on the factorial plane offered an overall view of the relationships between the themes from the interviews.

The first quadrant (top right), the cluster named ‘Meaning of being a migrant’, was represented by words in green. It contained keywords reflecting responses to the question ‘What does it mean for you to be a migrant?’ Keywords such as *being, migrant, world, idea, life, Germany, recognize, long, story, understand, country, return, leave, parent* and *language* focused on the migrant's identity, personal experiences, and challenges associated with moving to a new country. These terms emphasized the essence of being a migrant and highlighted the importance of understanding and recognizing the cultural and personal struggles that migrants face. Professionals often expressed empathy towards migrants, sometimes drawing from their own experiences as migrants themselves and facing integration challenges. This personal connection established a welcoming and understanding environment within the centre.

The second quadrant (top left) of the cluster named ‘Challenges and strengths in the reception centre’ was represented by the red colour. It included responses to the question ‘What are the strengths and challenges of your work in Camini?’ Keywords such as *handle, beneficiary, model, integration, hospital, community, doctor, medical, activity, document, directly, resource, project, coordinate, role, condition* and *request* reflected the practical aspects of resource management, documentation, project coordination, and integration of migrants into the community. The professionals encountered significant challenges, such as territorial barriers limiting access to services and the lack of hospital facilities hindering adequate medical care. Despite these difficulties, they recognized strengths, such as the ability to create effective integration models and manage community projects that supported and provided resources to migrants.

Between the first and second quadrants, the light blue cluster was called ‘Suggestions for changes and improvements’. It encompassed responses to the question ‘If you could change something, what would you change, and what would you keep as it is?’ Terms such as *change, implementation and interventions* indicated a willingness to introduce new measures and practices to better meet migrants' needs. A key area for improvement was health services, with a strong need for more accessible and adequate healthcare, highlighted by the words: *health* and *need*. The urgency to adapt the centre's responses to migrants' specific needs was indicated by the words *respond* and *need*. Furthermore, professionals emphasized maintaining certain effective aspects of the centre, such as the integration model that facilitated migrants' inclusion into the local community. The cluster's position between the two quadrants signified its influence from both reflections on migrants' identity and the practical operations of the centre. The fourth quadrant (bottom right) of the cluster, called ‘Emotional experiences’ was represented by purple words. It included responses to the question ‘What inspired you to start this journey, and can you share a significant experience from your time in Camini that shaped your perspective or approach?’ Keywords such as *experience, negative, positive, respect, moment, woman, listen, talk, difficulty, remember, know, word, element*, and *external* highlighted the mix of positive and negative emotional experiences professionals encountered. Significant episodes, such as expressions of gratitude from migrants or overcoming difficulties through respect and mutual understanding, left them notable impacts. However, negative episodes, such as difficulties, conflicts or misunderstandings, are reported to need strong listening and communication skills to resolve. These experiences underscored the importance of interpersonal relationships and empathy communication in the work context.

The analysis identified two main factors. The first, explaining 50.29% of the variance, included migrant identity and integration (right semiaxes) with the operational aspects of the centre (left semiaxes). The right semiaxes emphasize the personal and cultural dimensions of migrants' experiences, while the left semiaxes highlighted the practical and managerial challenges of running the centre. Professionals must balance empathy for migrants' identities with effective resource management and operational efficiency. The second factor, covering 28.23% of the variance, included proposed changes with the emotional and relational experiences of professionals. The upper semiaxes focused on necessary improvements to the centre's services, particularly for migrants' health and integration. The lower semiaxes reflected the emotional challenges and interpersonal dynamics professionals face, underscoring the importance of effective and empathetic communication.

#### 3.3.2 Hierarchical Descendant Classification

**FIGURE 2:**
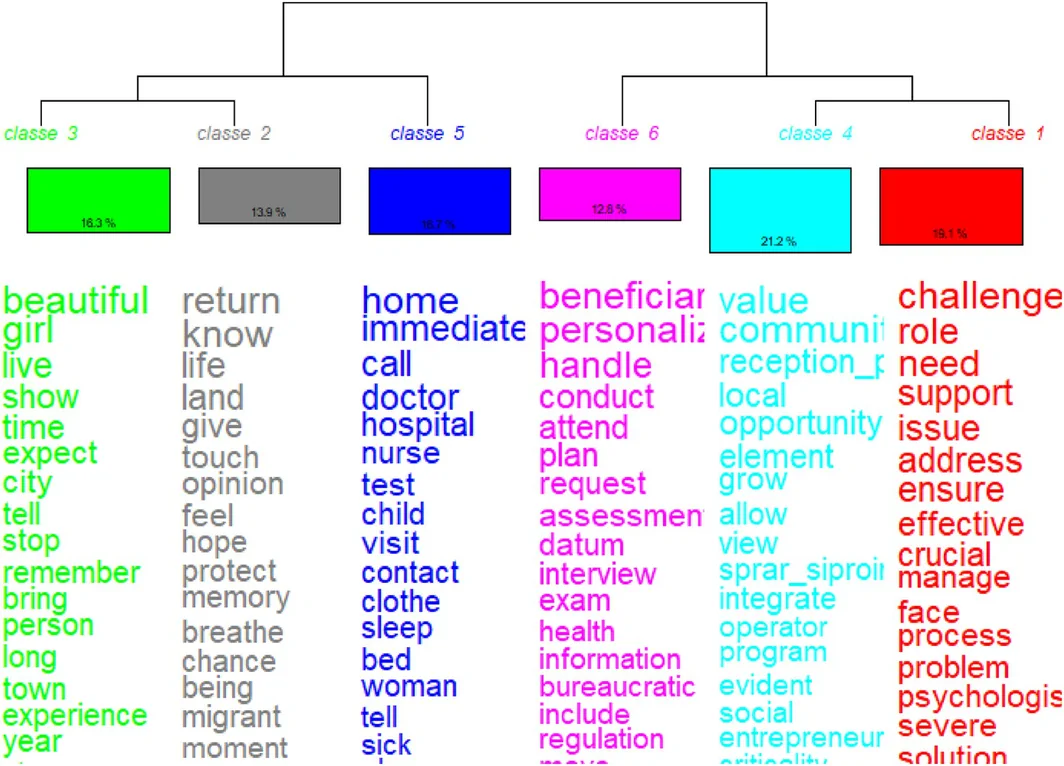
Hierarchical descendant classification.

The analysis of the interviews conducted with professionals working in the reception centre identified six main thematic clusters, each representing a specific dimension of their work and experiences (Figure 2).

Cluster 1 (19.1%), in red, called ‘Addressing complex challenges’, captured the professionals' focus on overcoming significant barriers and ensuring effective outcomes. Words like *challenge, role, need, support, issue, address, ensure, effective, crucial, process, problem, severe, solution* emphasized the strategic and critical thinking required to navigate operational difficulties, address systemic barriers, and support migrants in high‐stakes situations. Cluster 2 (13.9%), in grey, was called ‘Personal reflections on migration’, and represented the emotional and reflective dimension of migration. Words such as *return, know, life, land, give, touch, protect, memory, breathe, chance, being, migrant, moment* suggested a focus on understanding the migrant's journey, the emotional weight of displacement, and the need for empathy in their roles. This cluster highlighted the personal connections professionals develop with the concept of migration. Cluster 3 (16.3%), in green, was called ‘Significant experiences and interactions’ and it focused on the emotional rewards of professionals' work. Words like *beautiful, girl, live, show, time, expect, city, tell, stop, remember, bring, person, long, town, experience, year* reflected the joy and inspiration derived from positive interactions and transformative moments with migrants, as well as the cultural richness encountered in their roles. Cluster 4 (21.2%), in light blue, called ‘Community values’ was centred on the strengths of the reception system, including fostering community bonds and enabling personal and collective growth. Words such as *value, community, reception, local, opportunity, element, grow, allow, view, integrate, operator, program, evident, social, entrepreneur* emphasize the importance of building inclusive, supportive environments that empower both migrants and the local community. Cluster 5 (16.7%), in blue, called ‘primary assistance’, highlighted the hands‐on, caregiving side of professionals' work. Words such as *home, immediate, call, doctor, hospital, nurse, test, child, visit, contact, clothe, sleep, bed, woman, tell, sick* reflect the professionals' focus on addressing migrants' urgent needs, including medical care, housing, and emotional well‐being. This cluster illustrated professionals' role as first responders to the immediate challenges migrants face upon arrival. Cluster 6 (12.8%), in violet, was called ‘Managing individual needs’ reflected the balance between individualized care and navigating institutional processes. Words like *beneficiary, personalization, handle, conduct, attend, plan, request, assessment, interview, exam, health, information, bureaucratic, include, regulation* show the professionals' efforts to tailor their support to each migrant while managing bureaucratic and regulatory constraints.

#### 3.3.3 Principal Component Analysis

**FIGURE 3:**
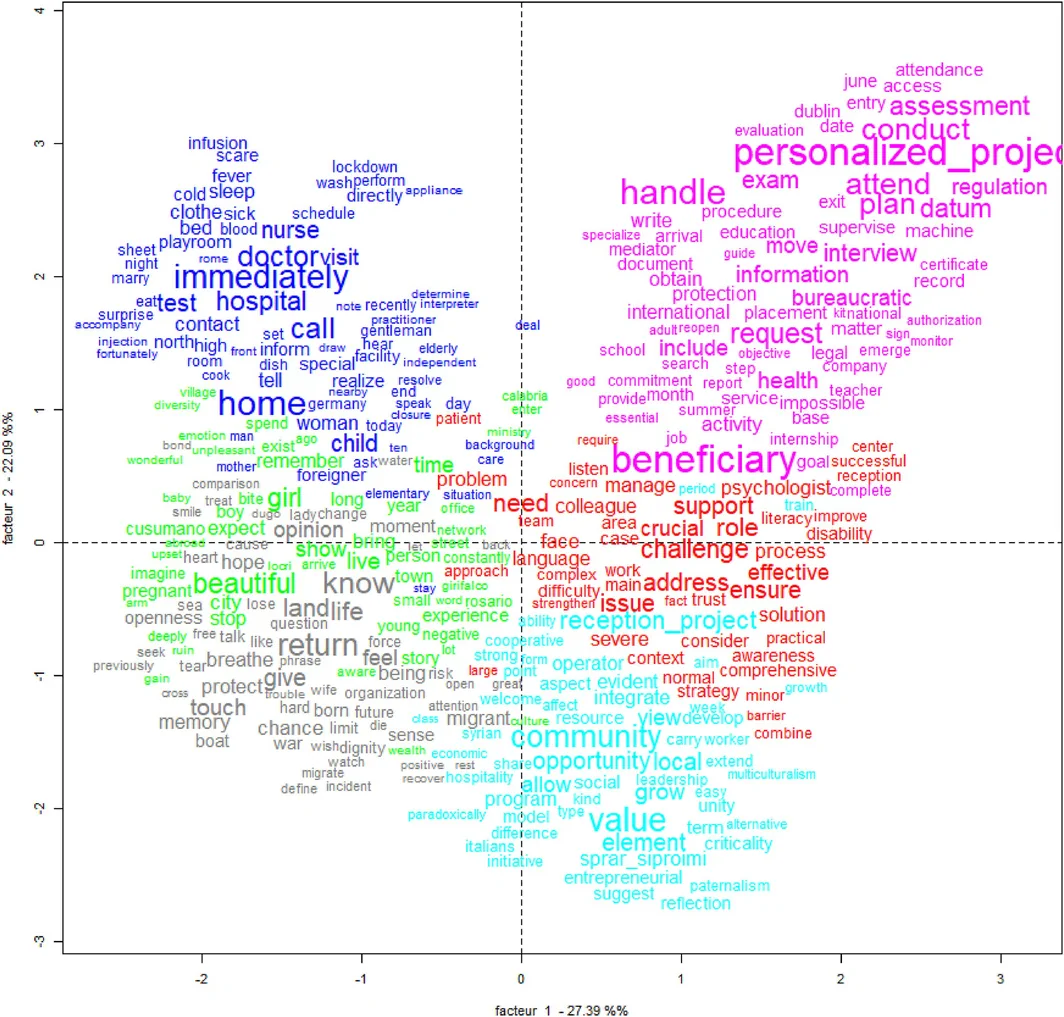
Principal component analysis.

The Principal Component Analysis (PCA) (Figure 3) revealed the relationships between six clusters by placing them on a factorial plane, showing both thematic similarities and differences. This analysis helped understand the roles of professionals, highlighting the balance they must maintain between systemic demands and the human, emotional connections central to their work in reception centres.

The clusters were grouped into two main thematic areas. The upper side focused on systemic management, bureaucratic challenges, and immediate care, where institutional processes met urgent needs. The lower side emphasized personal experiences, emotional connections, and long‐term community integration goals. On the right, clusters were related to strategic and systemic approaches, while the left highlighted human‐centred, reflective experiences. This distribution demonstrated the dual nature of professionals' work, balancing immediate assistance with broader integration efforts.

The first component (Factor 1, horizontal axis—27.39%) was called ‘Professional Duality’. It reflected the tension between two key aspects of professionals' roles. On the positive side, ‘Systemic Responsibility’ focused on managing bureaucratic complexities and operational tasks. On the negative side, ‘Personal Engagement’ highlighted emotional involvement, human connections, and introspective reflections, capturing the need for professionals to balance efficiency with empathy.

The second component (Factor 2, vertical axis—22.09%) was named ‘Temporal Priorities’. It illustrated the need for professionals to balance immediate and long‐term responsibilities. The positive side, ‘Immediate Response’, focused on addressing urgent needs like healthcare and safety. The negative side, ‘Sustainable Integration’, emphasized long‐term efforts to foster inclusion and build community relationships. This factor showcased the tension between short‐term actions and future‐oriented goals.

## 4 Discussion

This study provided a comprehensive analysis of the experiences, strengths, challenges, and potential improvements of healthcare, socio‐educational, and managerial professionals in managing the complex needs of VM within a migrant reception centre in southern Italy. The findings illustrated the multifaceted demands of working in such settings, requiring professionals to navigate complex operational frameworks while addressing the unique human and cultural needs of migrants.

The most important finding is the complex nature of professionals' work lying at the core of professionals' experience. On one hand, their dual role involves balancing systemic responsibilities, such as bureaucratic processes, operational organization, and the provision of healthcare and safety, with emotional and personal engagement. This engagement entails building meaningful connections with migrants while recognizing and valuing their stories and identities. On the other hand, temporal priorities emerged, reflecting the need to balance immediate needs, such as healthcare, housing, and safety, with long‐term goals of fostering integration through inclusive and sustainable strategies. These findings align with research illustrating the inherent tension between institutional efficiency and personalized care (Figura et al. 2024). This tension requires professionals to continuously adapt their approaches, balancing operational demands with empathy and individualized care to meet both institutional expectations and the complex needs of migrants (Arcadi et al. 2024; Gaya‐Sancho et al. 2021). While this duality is critical to their effectiveness, it also presents significant challenges, including stress and emotional fatigue, often exacerbated by insufficient support systems (Priebe et al. 2011; Roberts et al. 2021), requiring foresight, flexibility, and the ability to align institutional objectives with the holistic needs of migrants (Kennedy et al. 2021). Recent studies emphasized the necessity for both short‐ and long‐term adaptability in professional practices within reception centres addressing immediate priorities, such as healthcare, housing, and safety, while simultaneously working towards long‐term objectives of integration through inclusive and sustainable strategies (Roberts et al. 2021).

Resilience and adaptability, the multidisciplinary approach, the connections with local communities and the multifaced integration model emerged as key strengths. Resilience and adaptability, reported by professionals, help in deriving personal satisfaction from their work and reinforce their intrinsic motivation and sense of purpose. Multidisciplinary teams, rooted in a combination of technical expertise and human sensitivity, allow for cohesive and integrated responses to the complex and diverse needs of migrants. Literature highlights how the resilience and adaptability of multidisciplinary teams can foster integrated responses to the complex needs of migrants, emphasizing the importance of learning in enhancing the effectiveness of integrated health and social care teams (Bubendorff 2019; McCray et al. 2016). Additionally, connections with local communities and the multifaced integration model facilitated social inclusion and strengthened relationships between migrants and host communities, supporting migrants during their transitions. According to Peñuela‐O'Brien et al. (2023), it is crucial to adopt collaborative and inclusive approaches in promoting migrant integration. Gemignani and Giliberto (2021) and Driel and Verkuyten (2022) highlighted how active engagement with local communities amplifies positive outcomes for both migrants and host populations in rural countries.

The most crucial challenges reported by professionals include systemic barriers and individual challenges. Concerning systemic barriers, inadequate resources and bureaucratic complexities hinder the timely delivery of care and essential services and, in rural areas, the lack of infrastructure, such as hospitals and medical centres, exacerbates these difficulties. The literature supports these findings, with Suphanchaimat et al. (2015) emphasizing how healthcare professionals often struggle to balance professional ethics with laws restricting migrants' access to care, and Mammana, Milani, et al. (2020) and Driel and Verkuyten (2022) discussing the disparities related to decentralized healthcare system, service fragmentation and access to local services which can further complicating providing care. To address these challenges, literature employ various strategies, such as collaborating with voluntary organizations and migrant communities and implementing intersectoral actions to improve migrants' healthcare access and social integration (Mammana, Gherardi, et al. 2020; Mammana, Milani, et al. 2020; Peñuela‐O'Brien et al. 2023). On an individual level, the emotional burden of interacting with vulnerable migrants, often marked by traumatic experiences, contributes to secondary trauma and burnout. In this regard, literature suggest the existence of identified three professional profiles: overburdened, idealizing, and engaged, among workers in refugee centres, emphasizing the importance of the emotional side of their roles (De Leo et al. 2021). Moreover, despite positive relationships with care recipients, significant racial and cultural tensions within workplace dynamics may still arise (Timonen and Doyle 2010). These tensions, coupled with cultural and linguistic barriers, pose significant obstacles, requiring advanced intercultural communication skills and culturally competent care strategies to address them. Priebe et al. (2011) emphasized the importance of psychological support to mitigate emotional stress and prevent burnout among professionals.

Concerning areas for improvement, professionals identified several opportunities for better addressing the needs of vulnerable migrants. Key priorities included strengthening healthcare services and connection between professionals. They also emphasized the importance of targeted training programs focusing on intercultural competencies, stress management, and strategies to overcome language barriers. Additionally, enhancing social integration through community‐based models and fostering stronger connections between migrants and local communities were also deemed essential. The literature supports these priorities, emphasizing the need for targeted interventions. Kennedy et al. (2021) highlighted the importance of enhancing professionals' capabilities in migration contexts, while Peñuela‐O'Brien et al. (2023) recommend adopting intersectoral and person‐centred approaches to improve service access and promote social integration. Strategies such as adopting innovative technological solutions such as telehealth and simplifying bureaucratic procedures, particularly in rural areas, have been shown to improve service delivery (De Luca et al. 2021). Recommendations from the literature also include increased professional training, the adoption of holistic care approaches, and the implementation of intersectoral actions to improve healthcare access and social inclusion for migrants (Mammana, Gherardi, et al. 2020; Mammana, Milani, et al. 2020; Peñuela‐O'Brien et al. 2023).

The role of nurses in reception centres is particularly significant as they often represent the first point of healthcare contact for migrants. Leveraging their transversal skills, nurses integrate clinical care with cultural sensitivity, providing holistic support that considers migrants' traumatic experiences and specific needs. This transformative role may include managing health emergencies, coordinating local resources, and delivering health education to promote beneficiary autonomy. To enhance this capacity, specific strategies are essential, including continuous education programs focused on transcultural care, stress management, and emotional support. Additionally, involving nurses in the development of policies and protocols for reception centres can improve operational efficiency and care experiences. Investments in tools like telemedicine and language training can further empower nurses to deliver high‐quality care even in resource‐limited settings.

### 4.1 Strengths and Limitations

This study advances research by exploring the experiences, strengths, and challenges of professionals managing VM needs in stressful reception centre environments. It redefines their role as mediators between systemic demands and human‐centred care, emphasizing its relational and transformative nature beyond logistical tasks. Findings highlight how multidisciplinary collaboration fosters integrated responses addressing both immediate needs and long‐term inclusion. The study also underscores the human dimensions of care, where empathy, cultural sensitivity, and adaptability are as vital as operational efficiency.

Employing innovative analytical methods such as MCA and PCA, this research reveals hidden patterns in professionals' experiences, capturing the complexity of their roles. Their work requires balancing systemic responsibilities, emotional engagement, and the interplay of short‐ and long‐term goals, demonstrating the necessity of targeted support systems to enhance both their effectiveness and well‐being.

Although the findings are rooted in the Italian rural context, they offer analytically transferable insights into professional experiences that may be relevant to similar reception settings, where the tension between immediate responses and sustainable integration represents a shared and global challenge.

The multidimensional analysis model can be adapted to different healthcare systems, considering factors like service integration, transcultural training, and technological resources.

Despite its strengths, this study has limitations that warrant consideration.

A significant limitation of this study is the limited representation of nurses in the sample, which included only one participant from this profession. This underrepresentation restricts the ability to deeply explore the specific contributions of nurses and their unique experiences within multidisciplinary teams in reception centres. As nurses play a crucial role in managing migrants' healthcare needs, future research should aim to include a larger sample of nursing professionals to better understand their challenges and strategies. Moreover, future studies could examine how the nursing role interacts with other professional figures in multidisciplinary teams, providing a more comprehensive view of collaborative dynamics. A focus on different contexts, such as urban versus rural reception centres, could reveal further variations in nursing experiences and training needs.

### 4.2 Relevance for Clinical Practice

This study highlighted several important implications for clinical practice. First, professionals in reception centres require targeted support to navigate both systemic and relational demands effectively. Training programs should be developed to enhance their ability to manage bureaucratic complexities, address resource constraints, and build resilience against the emotional challenges of their work. These programs should include strategies for effective communication and self‐care to mitigate the risks of burnout, while also fostering interpersonal skills that strengthen relationships with migrants (Brewer et al. 2020). Additionally, improving healthcare delivery in reception centres is crucial, particularly in rural settings where infrastructure is often limited. Investments in telemedicine, specialized healthcare services, and improved access to medical facilities are essential to address current gaps (Okoli et al. 2025; Theodosopoulos et al. 2024).

From a research perspective, this study demonstrates the potential of advanced qualitative methodologies, such as AATD, to uncover latent dimensions and complex relationships in professionals' experiences. Future studies should continue to leverage these tools to deepen understanding and explore professional dynamics across diverse contexts (Sanip 2020). Moreover, comparative research that examines reception dynamics in urban versus rural centres or larger versus smaller facilities could further clarify how environmental factors influence professionals' roles and challenges. Incorporating the perspectives of other stakeholders, including migrants, policymakers, and community leaders, would provide a more holistic view of the systemic and interpersonal dynamics at play (Figura et al. 2024). Longitudinal research examining the long‐term effects of dual demands on professionals' well‐being and retention would also be valuable for developing sustainable workforce strategies (Al‐Btoush and El‐Bcheraoui 2024).

In terms of health policy, the findings emphasize the need for systemic changes to alleviate the pressures on reception centres and their staff. Streamlining bureaucratic processes could reduce administrative burdens and improve operational efficiency, while increased funding for healthcare infrastructure in rural areas is vital to ensure equitable access to services (Mancini et al. 2019). Policies that promote collaborative integration models, involving professionals, local communities, and migrants, could foster inclusion and mutual growth (Okoli et al. 2025). Mental health support for professionals should also be prioritized, with accessible counselling and peer support networks to address the emotional toll of their work (Falgas‐Bague et al. 2025). By addressing these systemic challenges and supporting the workforce, health policies can enhance the quality of care provided to migrants while ensuring the sustainability and well‐being of the professionals who deliver these essential services.

## 5 Conclusion

This study explored the lived experiences of professionals working in an Italian rural reception centre caring for vulnerable migrants, highlighting the complex interplay between systemic responsibilities and personal engagement in everyday practice. The findings illustrate how professionals navigate competing demands related to immediate assistance, emotional involvement, and long‐term integration goals, within contexts characterized by limited resources and organizational constraints.

By adopting a qualitative approach based on multidimensional textual analysis, the study provides an in‐depth understanding of how these experiences are constructed and negotiated, offering analytically transferable insights into similar reception settings where comparable tensions are present. Rather than aiming for direct generalization, the findings should be interpreted in relation to the specific contextual features of rural reception systems.

Overall, this study underscores the importance of supporting professionals working with vulnerable migrant populations through organizational strategies, targeted training and attention to professional well‐being, as discussed in relation to existing evidence. Addressing these aspects is essential to sustain the workforce and to promote the quality and continuity of care in migrant reception contexts.

## Author Contributions

Conceptualization: M.F., P.A., G.P., S.S., I.U., E.V., R.A. Data curation: M.F., P.A., G.P., S.S. Formal analysis: M.F. Funding acquisition: I.U., E.V., R.A. Investigation: M.F., P.A., G.P., S.S. Methodology: M.F., P.A. Project administration; E.V., R.A. Software: M.F. Resources: M.F. Supervision: E.V., R.A. Validation: G.P., S.S., I.U., E.V., R.A. Visualization: M.F., P.A. Writing – original draft: M.F., P.A. Writing – review and editing: G.P., S.S., I.U., E.V., R.A.

## Funding

This study was funded by the Center of Excellence for Nursing Scholarship, Rome, Italy. This article was published in open access thanks to funding from Worklaw Medical University, grant Subz. L010.25.053.

## Conflicts of Interest

The authors declare no conflicts of interest.

## Supporting information


**Data S1:** COnsolidated criteria for REporting Qualitative research (COREQ): 32‐item checklist.

**Figure S1:** Fraire’s EMDA 7‐steps model.
**Table S1:** Metadata lines.

## Data Availability

The data that support the findings of this study are available from the corresponding author upon reasonable request.
