# Supplementary material for: Exploring Professional Experiences in Caring for Vulnerable Migrants in an Italian Rural Reception Centre: A Qualitative Study Using Multidimensional Textual Analysis—Professional Experiences in Rural Migrant Care
Source: Nurs Open. 2026 Aug 30;13(9):e70757. doi: 10.1002/nop2.70757 (PMC13526467; doi:10.1002/nop2.70757)
Supplement: Supplementary file 1 [file NOP2-13-e70757-s001.docx]

**SUPPLEMENTARY MATERIAL**

Figure S 1: Fraire's EMDA 7-steps model

| *parl_resp | *sett_org | *theme_exp |
| --- | --- | --- |
| *parl_resp | *sett_org | *theme_change |
| *parl_resp | *sett_org | *theme_beingmigr |
| *parl_coord | *sett_org | *theme_activity |
| *parl_coord | *sett_org | *theme_exp |
| *parl_coord | *sett_org | *theme_change |
| *parl_coord | *sett_org | *theme_beingmigr |
| *parl_opleg | *sett_org | *theme_activity |
| *parl_opleg | *sett_org | *theme_exp |
| *parl_opleg | *sett_org | *theme_change |
| *parl_opleg | *sett_org | *theme_beingmigr |
| *parl_opinsprof | *sett_org | *theme_activity |
| *parl_opinsprof | *sett_org | *theme_exp |
| *parl_opinsprof | *sett_org | *theme_change |
| *parl_opinsprof | *sett_org | *theme_beingmigr |
| *parl_medcult | *sett_soc | *theme_activity |
| *parl_medcult | *sett_soc | *theme_exp |
| *parl_medcult | *sett_soc | *theme_change |
| *parl_medcult | *sett_soc | *theme_beingmigr |
| *parl_teacher | *sett_soc | *theme_activity |
| *parl_teacher | *sett_soc | *theme_exp |
| *parl_teacher | *sett_soc | *theme_change |
| *parl_teacher | *sett_soc | *theme_beingmigr |
| *parl_oss | *sett_soc | *theme_activity |
| *parl_oss | *sett_soc | *theme_exp |
| *parl_oss | *sett_soc | *theme_change |
| *parl_oss | *sett_soc | *theme_beingmigr |
| *parl_profed | *sett_soc | *theme_activity |
| *parl_profed | *sett_soc | *theme_exp |
| *parl_profed | *sett_soc | *theme_change |
| *parl_profed | *sett_soc | *theme_beingmigr |
| *parl_socassist | *sett_soc | *theme_activity |
| *parl_socassist | *sett_soc | *theme_exp |
| *parl_socassist | *sett_soc | *theme_change |
| *parl_socassist | *sett_soc | *theme_beingmigr |
| *parl_liuth | *sett_soc | *theme_activity |
| *parl_liuth | *sett_soc | *theme_exp |
| *parl_liuth | *sett_soc | *theme_change |
| *parl_liuth | *sett_soc | *theme_beingmigr |
| *parl_nurse | *sett_healthcare | *theme_activity |
| *parl_nurse | *sett_healthcare | *theme_exp |
| *parl_nurse | *sett_healthcare | *theme_change |
| *parl_nurse | *sett_healthcare | *theme_beingmigr |
| *parl_doctw | *sett_healthcare | *theme_activity |
| *parl_doctw | *sett_healthcare | *theme_exp |
| *parl_doctw | *sett_healthcare | *theme_change |
| *parl_doctw | *sett_healthcare | *theme_beingmigr |
| *parl_doctm | *sett_healthcare | *theme_activity |
| *parl_doctm | *sett_healthcare | *theme_exp |
| *parl_doctm | *sett_healthcare | *theme_change |
| *parl_doctm | *sett_healthcare | *theme_beingmigr |
| *parl_psysup | *sett_healthcare | *theme_activity |
| *parl_psysup | *sett_healthcare | *theme_change |
| *parl_psysup | *sett_healthcare | *theme_beingmigr |
| *parl_etnopsy | *sett_healthcare | *theme_activity |
| *parl_etnopsy | *sett_healthcare | *theme_exp |
| *parl_etnopsy | *sett_healthcare | *theme_change |
| *parl_etnopsy | *sett_healthcare | *theme_beingmigr |
| *parl_psy | *sett_healthcare | *theme_activity |
| *parl_psy | *sett_healthcare | *theme_exp |
| *parl_psy | *sett_healthcare | *theme_change |
| *parl_psy | *sett_healthcare | *theme_beingmigr |

Table S 1: metadata lines
